# Autoantibodies to Tumor-Associated Antigens in Breast Carcinoma

**DOI:** 10.1155/2010/264926

**Published:** 2010-11-21

**Authors:** Ettie Piura, Benjamin Piura

**Affiliations:** ^1^Department of Obstetrics and Gynecology, Sapir Medical Center, Sackler School of Medicine, University of Tel-Aviv, Kfar-Saba 44281, Israel; ^2^Unit of Gynecologic Oncology, Department of Obstetrics and Gynecology, Soroka Medical Center and Faculty of Health Sciences, Ben-Gurion University of the Negev, Beer-Sheva 84101, Israel

## Abstract

Autoantibodies (AAbs) to tumor-associated antigens (TAAs) have been identified in the circulation of patients with cancer. This paper will focus on recent knowledge related to circulating AAbs to TAAs in breast carcinoma. So far, the following TAAs have been identified to elicit circulating AAbs in breast carcinoma: p53, MUC-1, heat shock proteins (HSP-27, HSP-60, and HSP-90), HER2/neu/c-erb B2, GIPC-1, c-myc, c-myb, cancer-testis antigens (NY-ESO-1), BRCA1, BRCA2, endostatin, lipophilin B, cyclin B1, cyclin D1, fibulin, insulin-like growth factor binding protein 2 (IGFBP-2), topoisomerase II alpha (TOPO2*α*), and cathepsin D. Measurement of serum AAbs to one specific TAA only is of little value for screening and early diagnosis of breast carcinoma; however, assessment of AAbs to a panel of TAAs may have promising diagnostic potential.

## 1. Introduction

The development of circulating autoantibodies (AAbs) to tumor-associated antigens (TAAs) has been observed to be associated with cancer [1, 2]. Unlike traditional tumor markers (e.g., CA-15-3, CA-19-9, CA-125, and CEA), which are soluble proteins shed by bulky tumors, serum AAbs to TAAs are detectable even when the tumor is very small [2]. Thus, the identification of AAbs to TAAs could potentially be used as a novel tool for screening and early diagnosis of cancer [2–6]. Sahin et al. [7] introduced in 1995 a method called SEREX (serological analysis of recombinant cDNA expression libraries) that has broad applicability to the analysis of the humoral immune response to cancer. Originally, they had used mRNA isolated from tumor tissue with the assumption that specific TAAs could be isolated. This has turned out to be incorrect, since there is no such thing as a TAA that is only expressed in tumors. Thus, the SEREX method as described by the original authors has been highly modified and uses nowadays cDNA libraries from a variety of cell lines and not just from tumor tissues. So far, over 2,000 candidate TAAs in many types of human cancer have been identified and separated into six categories [5, 8–11]: (1) differentiation antigens (expressed by cancers and a restricted subset of normal cells, e.g., tyrosinase, melan-A/MART-1, NY-BR-1, and gp100), (2) mutational antigens (e.g., CDK4, *β*-catenin, caspase-8, and p53), (3) amplification (overexpression) antigens (e.g., c-erb B2/HER2/neu, NY-C0-58, and p53), (4) splice variant antigens (e.g., NY-CO-37/PDZ-45, and ING1), (5) viral antigens (e.g., HPV and EBV), and cancer-testis (CT) antigens (e.g., NY-ESO-1, MAGE-A, and LAGE-1). The humoral immune response elicited by TAAs could have two major clinical applications [9]: (1) AAbs to TAAs could represent novel biomarkers for cancer diagnosis, prognosis, monitoring, and prediction of response to chemotherapy, (2) TAAs might be used as targets for immunotherapy of cancer. Notwithstanding, efforts to predict cancer based on autoimmunity to either an individual TAA or even tailor-made panel of TAAs have not yet resulted in serologic biomarkers with definitive predicting specificity and sensitivity [12]. It has, however, been shown by some investigators that the use of tailor-made panel of TAAs, rather than individual TAAs, enhances the likelihood of detecting cancer-associated AAbs with potential diagnostic value [3, 12].

In the USA, breast carcinoma is diagnosed in approximately 193,000 women and 2,000 men yearly with an age-adjusted incidence of 125 new cases/100,000 women/year and 1 new case/100,000 men/year, and it causes approximately 41,000 deaths (40,500 women and 500 men) each year [13, 14]. Breast carcinoma is the first most common cancer among women (27% of all cancers in women) and the second most common cause of death from cancer, after lung carcinoma, in women (15% of all cancer deaths in women) [13]. The National Cancer Institute (NCI) has estimated that 12.7% (1/8) of women born today in the USA will be diagnosed with breast carcinoma at some time in their lives [15]. The five-year survival rate overall of women with breast carcinoma in the USA is about 90% [14].

Worldwide, breast carcinoma is by far the most frequent cancer among women with an estimated 1.38 million new cases diagnosed in 2008 (23% of all malignancies in women) and ranks second overall (10.9%), after lung carcinoma, of all malignancies in both sexes [16]. The estimated incidence of breast carcinoma in 2008 worldwide has been 39 new cases/100,000 women. The incidence has been estimated to vary from 19.3 in Eastern Africa to 89.9 in Western Europe, and is high (greater than 80) in developed countries (except Japan) and low (less than 40) in most of the developing countries [16]. Breast carcinoma has been estimated to cause 458,000 deaths in 2008 worldwide (13.7% of all cancer deaths in women and 6% of all cancer deaths in both sexes). The estimated mortality from breast carcinoma in 2008 worldwide has been 12.5 deaths/100,000 women. Breast carcinoma is the most frequent cause of death from cancer in women worldwide and the fifth cause of death from cancer, after lung, stomach, liver, and colorectal carcinoma, in both sexes [16].

Current screening modalities for breast carcinoma diagnosis include mammography, ultrasound (US), and magnetic resonance imaging (MRI); however, there is still an urgent need to develop an alternative modality of screening for earlier diagnosis [17]. The use of serum-soluble tumor antigens, such as CA-15-3 glycoprotein, as biomarkers for detection of breast carcinoma has been limited by their insufficient specificity and sensitivity, particularly for organ confined early-stage disease. Consequently, CA-15-3 is not recommended for use in the screening or detection of breast carcinoma [18–21]. This is in part due to the elevation of CA-15-3 in benign conditions including breast, liver, and kidney disorders and other cancers [22]. Thus, there is a need to discover novel biomarkers, such as AAbs to specific breast carcinoma TAAs, for screening, early diagnosis, prediction of prognosis, and monitoring of treatment. There is also a need to develop new therapeutic approaches, such as immunotherapy, for the management of breast carcinoma. The establishment of AAbs to TAAs as biomarkers for breast carcinoma and the development of successful immunotherapeutic strategies require the identification and characterization of immunogenic breast carcinoma TAAs that will be recognized by the host immune system. Thus far, only few circulating AAbs to specific breast carcinoma TAAs have been identified and investigated. In breast carcinoma, like in other malignancies, the use of tailor-made panel of TAAs, rather than individual TAAs, enhances the likelihood of detecting cancer-associated AAbs with potential diagnostic value.

This paper will review the up-to-date knowledge related to AAbs against individual TAAs in breast carcinoma.  Table 1 shows the frequency of identified AAbs to breast carcinoma TAAs.

## 2. Autoantibodies to p53 Protein

The wild-type p53 gene is a tumor suppressor gene located on chromosome 17p13 and encodes a 53-kDa nuclear phosphoprotein that normally acts as a guardian of the integrity of the genome [27, 44, 45]. Mutations in p53 are the most common genetic changes found in human malignancies and the mutational status of p53 is prognostic in many malignancies [46]. In breast carcinoma, p53 mutations have been shown to be associated with worse overall and disease-free survival, independent of other risk factors, and have been implicated in resistance to anticancer therapies [47, 48]. Missense point mutations, which represent more than 85% of gene abnormalities, lead to a conformational change which stabilizes the p53 protein and allows it to accumulate in the nucleus to relatively high levels [27, 44, 45, 49–51]. Accumulation of the mutant p53 in tumor cells can elicit a humoral immune response leading to the production of anti-p53 AAbs [27]. Initially, it was thought that only tumors with missense p53 mutations resulting in p53 overexpression can elicit anti-p53 AAbs [27, 52–54]. Later on, however, anti-p53 AAbs have also been detected in sera from patients with tumors lacking p53 overexpression. Induction of anti-p53 AAbs in these patients might be due to the unusual presentation of large amounts of wild-type p53 from necrotic large tumors or metastases [27, 55]. Recently, it has been shown that anti-p53 AAbs are directed against immunodominant epitopes localized in the amino and carboxy terminal ends of the p53-protein, unrelated to the mutational hot spot [27, 51, 56–58]. Epithelial ovarian carcinomas have been regarded as a tumor entity associated with the highest frequency (13%–46%) of circulating anti-p53 AAbs [59]; nevertheless, breast carcinomas are also associated with a considerable incidence (2.8%–47.5%) of serum anti-p53 AAbs. Thus, breast carcinomas, alongside epithelial ovarian carcinomas, are among the most immunogenic malignancies inducing anti-p53 AAbs response. Indeed, while mutation of p53 appears a seminal event in carcinogenesis and is present in ~30% of breast carcinoma patients, it is still unclear why only a subset of p53 mutation-positive breast carcinoma patients (~50%) generates anti-p53 AAbs [27]. It has been suggested that only p53 mutations that are localized in exons 5 and 6 with an altered protein conformation and that bind to HSP-70 are associated with anti-p53 AAbs [54, 58]. Further studies, however, have demonstrated that other factors contribute to the humoral immune response to p53 protein and suggested that the capacity to elicit a humoral immune response is linked to the biological background of the patients [27, 52]. It is possible that for an identical mutation, the humoral immune response is dependent on the specific combination of MHC class I and II molecules expressed by each individual [27].

Dalifard et al. [26] demonstrated by ELISA in 1999 that anti-p53 AAbs were present in the sera of 8/101 (7.9%) patients with breast carcinoma. The presence of serum anti-p53 AAbs correlated neither with p53 cytosolic assay nor with prognostic factors. The authors concluded that serum anti-p53 AAb assay is not useful for the selection of patient groups with poor prognosis [26].

Soussi [27] surveyed the literature from 1979 through 1999 on anti-p53 AAbs in the sera of patients with various types of cancer. Serum anti-p53 AAbs were present in 1600/9489 (16.8%) patients with different malignancies and in 35/2404 (1.4%) healthy controls (*P* < 10^−4^). Fifteen studies [52, 54, 58, 60–71] examined anti-p53 AAbs in the sera of breast carcinoma patients. The frequency of anti-p53 AAbs in the sera of breast carcinoma patients ranged in these studies from 2.8% to 47.5%. Overall, the presence of anti-p53 AAbs was demonstrated in the sera of 296/2006 (14.7%) breast carcinoma patients [27]. *χ* ^2^ test showed that the frequency of anti-p53 AAbs was significantly higher in the sera of breast carcinoma patients compared to healthy controls (*P* < 10^−4^) [27]. Since serum anti-p53 AAbs are truly rare in the normal population, the specificity of this assay for the detection of breast carcinoma has been estimated to attain 95%. Nevertheless, since anti-p53 AAbs were present on the average in the sera of only 15% of breast carcinoma patients, the sensitivity of this assay for the detection of breast carcinoma has been estimated to reach only 30% [27]. Five studies [58, 60, 63, 65, 67] indicated that serum anti-p53 AAbs are found in patients with tumors that have high grades and/or that are negative for steroid hormone receptors, two clinical parameters known to be associated with p53 mutations and bad prognosis. Two studies [67, 68] found an association between serum anti-p53 AAbs and short survival whereas one study [71] did not find any association, and another study [66] found an association with good survival.

Regele et al. [24] demonstrated by ELISA in 2003 that serum anti-p53 AAbs were present in 11/24 (45.8%) patients at initial diagnosis of breast carcinoma. In seven of these 11 patients, therapy was paralleled by decreasing anti-p53 AAb titers; in four, relapse was preceded by an increase of the titer. Two patients, who initially tested negative, seroconverted to anti-p53 AAb positivity upon relapse. The authors [24] concluded that monitoring of serum anti-p53 AAbs during followup can be informative about the clinical course of the disease and the development of breast carcinoma relapse can be preceded by an increase of serum anti-p53 AAb titer.

Gao et al. [25] showed by ELISA in 2005 that serum anti-p53 AAbs were present in 31/144 (21.5%) patients with breast carcinoma and 12/242 (4.9%) healthy controls. The presence of serum anti-p53 AAbs was associated with several poor prognostic factors including higher clinical stage (*P* = .0233), lymph nodes metastasis (*P* = .0033), negative ER expression (*P* = .0250) and positive HER2/c-erbB-2 status (*P* = .0227). There was also a strong correlation between serum anti-p53 AAbs and tumor immunohistochemical positivity for p53 (*P* < .0001). The authors [25] speculated that serum anti-p53 AAbs could serve as a useful and convenient marker for the detection and prognosis of breast carcinoma.

Chapman et al. [23] showed by ELISA in 2007 that preoperative serum anti-p53 AAbs were present in 22/94 (23.4%) patients with newly diagnosed breast carcinoma and 6/40 (15%) patients with ductal carcinoma in situ. Positive seroreactivity was defined as an absorbance value greater than the mean plus two standard deviations of a normal cohort. The sensitivity and specificity were 24% and 96%, respectively, for breast carcinoma, and 15% and 96%, respectively, for ductal carcinoma in situ. The authors [23] concluded that measurement of serum AAbs to p53 protein only is of little value for screening and early diagnosis of breast carcinoma; however, AAbs to p53 may have promising diagnostic potential when incorporated in AAb assays against a panel of TAAs.

Lu et al. [1] demonstrated with ELISA in 2008 that AAbs to p53 protein were present in the sera of 22/220 (10%) breast carcinoma patients compared to 2/200 (1%) healthy controls. It has been concluded that AAbs to p53 have no diagnostic potential when examined alone; however, they may possibly have diagnostic potential when incorporated in AAB assays to a panel of TAAs.

## 3. Autoantibodies to MUC1 Protein

Polymorphic epithelial mucin (PEM, MUC1), a human mucin family member, is a high-molecular-weight (over 400 kDa) transmembrane glycoprotein. It is expressed in a hyperglycosylated form and low levels by many types of normal epithelial cells and in a hypoglycosylated form and high levels by most epithelial adenocarcinomas including breast and ovarian carcinoma [72, 73]. About one-quarter of breast and ovarian carcinoma patients have circulating AAbs to MUC1, either free or bound to immune complexes. While the presence of these immune complexes has prognostic significance in cancer patients, the significance of free AAbs to MUC1 is less clear [74]. AAbs to MUC1 have been described and correlated with a more favorable prognosis; thus, it seems that risk for breast carcinoma might be reduced by preexisting MUC1-specific immunity [75, 76].

Using ELISA, Kotera et al. [28] demonstrated in 1994 that anti-MUC1 AAbs were present in the sera of 2/24 (8.3%) breast carcinoma, 2/12 (16.7%) pancreatic carcinoma, and 1/10 (10%) colon carcinoma patients. Overall, the presence or absence of serum anti-MUC1 AAbs did not correlate with the levels of circulating mucin or stage of disease [28]. von Mensdorff-Pouilly et al. [29] demonstrated by sandwich enzyme-linked immunoassay in 1996 that anti-MUC1 AAbs were present in the sera of 2/96 (2.1%) healthy controls, 15/40 (37.5%) patients with benign breast tumor, 36/140 (25.7%) patients with early-stage breast carcinoma and 11/61 (18%) patients with advanced-stage breast carcinoma. Serum anti-MUC1 AAbs were elevated in 24/74 (32.4%) node-negative patients and in 12/59 (20.3%) node-positive patients and absolute values were higher in node-negative patients (*P* = .0168). There was an inverse correlation between positivity for serum anti-MUC1 AAbs and extent of disease; while 3/6 (50%) patients with a carcinoma in situ were positive, only 1/15 (6.7%) patients with more than five nodes involved had elevated levels of anti-MUC1 AAbs. All seven patients with distant metastases at first diagnosis were anti-MUC1 AAb-negative. Twenty-eight of 133 patients had a recurrence during followup; 23 (82%) of these 28 patients were anti-MUC1 AAb-negative at the time of first diagnosis. The 5-year survival of 13 patients who had elevated pretreatment serum levels of CA-15-3 (>30 U/mL) and were anti-MUC1 AAb-positive was better than the 5-year survival of 41 patients who had elevated pretreatment serum levels of CA-15-3 and were anti-MUC1 AAb-negative (100% versus 71%, *P* = .0457). The authors [29] suggested that a natural humoral immune response to MUC1 seems to protect against disease progression, while lack of immune reaction, or immune tolerance developing in the course of disease, could be an additional risk factor more frequently associated with an unfavorable outcome.

Chapman et al. [23] showed by ELISA in 2007 that serum anti-MUC1 AAbs were present in 19/94 (20.2%) patients with newly diagnosed breast carcinoma and 10/40 (25%) patients with ductal carcinoma in situ. Positive seroreactivity was defined as an absorbance value greater than the mean plus two standard deviations of a normal cohort. The sensitivity and specificity were 20% and 98%, respectively, for breast carcinoma, and 23% and 98%, respectively, for ductal carcinoma in situ. The authors [23] concluded that measurement of serum AAbs to MUC1 protein only is of little value for screening and early diagnosis of breast carcinoma; however, AAbs to MUC1 may have promising diagnostic potential when incorporated in AAb assays against a panel of TAAs.

Lu at al. [1] observed an increased AAb response to p53, HER-2, MUC1, topoisomerase II alpha (TOPO2*α*), insulin-like growth factor binding protein 2 (IGFBP2), cyclin D1, and Cathepsin D in breast carcinoma patients. Nonetheless, the most frequently encountered AAb response was directed against MUC1 protein, which was detected in 20/100 (20%) breast carcinoma patients compared to 3/100 (3%) healthy controls [1].

Obviously, serum AAb-assay against MUC1 protein only is of little value for screening and early diagnosis of breast carcinoma; however, AAbs to MUC1 may have promising diagnostic potential when incorporated in AAb assays against a panel of TAAs. Moreover, there is support for the notion that preexisting AAbs to MUC1 may reduce the risk of developing breast carcinoma and presence of AAbs to MUC1 in breast carcinoma is correlated with a more favorable prognosis.

## 4. Autoantibodies to Heat-Shock Proteins

Heat-shock proteins (HSPs) are cytoplasmic proteins that act as molecular chaperones for protein molecules in various intracellular processes. They play an important role in protein-protein interactions, including folding and conformation and prevention of inappropriate protein aggregation. They are called heat-shock proteins since they were first discovered in cells exposed to high temperatures. However, their synthesis is also accentuated under other stress conditions, such as exposure of the cell to inflammation, infection, ischemia, toxins, cytotoxic drugs, and malignant transformation. HSPs have been classified into families according to their molecular weight [77–79].

Overexpression of HSP-27 in breast carcinoma has been associated with shorter disease-free survival [30, 80, 81]. Conroy et al. [30] demonstrated by ELISA in 1998 that serum AAbs to HSP-27 were present in 219/579 (37.8%) breast carcinoma patients compared to 1/53 (1/9%) healthy subjects (*P* < .001). The mortality rate was lower in women with AAbs to HSP-27 than in those who lacked such AAbs (*P* = .006). Thus, a significant association has been found between the presence of serum AAbs to HSP-27 and improved survival in breast carcinoma patients [30].

HSP-60 is an abundant, highly conserved protein mostly localized in the mitochondrial matrix. It plays a role in the regulation of various cellular functions and assists in mitochondrial protein folding, unfolding, and degradation. HSP-60 is involved also in the process of apoptosis and interacts with proteins implicated in cell cycle regulation [31]. With use of ELISA, Desmetz et al. [31] demonstrated the presence of serum AAbs to HSP-60 (seropositivity was defined as a value greater than the mean of the normal population plus two standard deviations) in 16/49 (32.6%) ductal carcinoma *in situ* (DCIS) patients and 18/58 (31%) early-stage breast carcinoma patients compared to 4/93 (4.3%) healthy subjects. This corresponded to a significant difference between DCIS patients (*P* < .0001) or early-stage breast carcinoma patients (*P* < .0001) and healthy controls. The frequency of AAbs to HSP-60 was significantly higher in high-grade DCIS patients (11/23, 47.8%) compared to low-grade DCIS patients (5/26, 19.2%) (*P* = .0188). This corresponded to a higher significant difference between high-grade DCIS patients and healthy controls (*P* < .0001) than between low-grade DCIS patients and healthy controls (*P* = .0233) [31]. The diagnostic performance (discriminating between breast carcinoma patients and healthy subjects) of AAbs to HSP-60 was represented by a specificity of 95.7%, sensitivity of 31.8%, positive predictive value of 89.5%, negative predictive value of 54.9%, and area under curve (AUC) of 63.7% [31]. Notably, no significant relation was found between the frequency of AAbs to HSP-60 and estrogen receptor (ER), progesterone receptor (PR) and HER-2 status [31]. It seems that measuring AAbs to HSP-60 has promising diagnostic potential for early detection of breast carcinoma; however, this is still inconclusive and further studies are needed. 

Conroy et al. [32] demonstrated by ELISA in 1995 that serum AAbs to HSP-90 were present in 46/125 (36.8%) breast carcinoma patients. Seropositivity was defined as a value greater than the mean value observed in normal controls plus three standard deviations. Multivariate analysis indicated that the presence of serum AAbs to HSP-90 (*P* < .04) and the presence of axillary lymph node invovlement (*P* < .001) correlated with the development of metastases [32]. Moreover, the presence of serum AAbs to HSP-90 correlated with the development of metastases even in patients without axillary lymph node involvement [32]. In 1998, Conroy et al. [33] showed by ELISA that serum AAbs to HSP-90 were present in 135/214 (63.1%) breast carcinoma patients before surgery and 127/200 (63.5%) breast carcinoma patients after surgery. Mortality rate from breast carcinoma was greater in women testing positive for AAbs to HSP-90 than those testing negative for AAbs to HSP-90. Although the difference between the two groups did not attest statistical significance, the authors have concluded that there appears to be an association between higher mortality rate from breast carcinoma and presence of serum AAbs to HSP-90 [33].

## 5. Autoantibodies to HER2/Neu/c-ErbB2 (p185) Protein

HER2/Neu/ErbB2 is a member of the epidermal growth factor receptor (EGFR) family that is amplified and overexpressed in 20%–30% of breast carcinomas. HER2-positive breast carcinoma yields a poor patient prognosis due to a high incidence of metastases and intrinsic resistance to endocrine and conventional chemotherapy. Treatments that target HER2 expression in cancer cells have been shown to be useful strategies to significantly reverse the malignancy induced by HER2 overexpression [82]. 

Serum anti-HER2 AAbs were detected by ELISA in 16/94 (17%) patients with newly diagnosed breast carcinoma and 5/40 (12.5%) patients with ductal carcinoma in situ [23]. Positive seroreactivity was defined as an absorbance value greater than the mean plus two standard deviations of a normal cohort. The sensitivity and specificity were 18% and 94%, respectively, for breast carcinoma, and 13% and 94%, respectively, for ductal carcinoma in situ. It has been concluded that measurement of serum AAbs to HER2 protein only is of little value for screening and early diagnosis of breast carcinoma; however, AAbs to HER2 may have promising diagnostic potential when incorporated in AAb assays against a panel of TAAs [23].

Disis et al. [34] demonstrated by western blot analysis in 1994 that serum anti-HER2 AAbs are present in 11/20 (55%) patients with breast carcinoma. They confirmed that the HER2 oncoprotein elicits an immune response and speculated that the HER2 oncoprotein may be used as a target for specific immunotherapy [34]. In 1997, Disis et al. [35] showed by capture enzyme-linked immunosorbent assay (ELISA) and verified by western blot analysis that serum anti-HER2 AAbs, at titers of > or = 1 : 100, were present in 12/107 (11.2%) early-stage breast carcinoma patients and in none (0%) of 200 healthy controls (*P* < .01). The presence of serum anti-HER2 AAbs correlated to overexpression of HER2 protein in the patient's primary tumor. Nine of 44 (20.4%) patients with HER2-positive tumor had serum anti-HER2 AAbs, whereas only 3/63 (4.8%) patients with HER2-negative tumor had serum anti-HER2 AAbs (*P* = .03). In contrast to the previous study, Disis et al. [16, 36] observed in 2000 that serum anti-HER2 AAbs were present in only 3/45 (6.6%) advanced-stage breast carcinoma patients. The lower incidence of serum anti-HER2 AAbs in advanced-stage disease compared to early-stage disease suggests that the humoral immune response to HER2 may have a role in limiting breast carcinoma progression.

Lu et al. [1] demonstrated by recombinant ELISA that AAbs to HER2 protein were present in the sera of 30/225 (13%) breast carcinoma patients compared to 10/200 (5%) healthy controls. It seems that serum AAbs to HER2 have no diagnostic potential when examined alone (unacceptable high false negative rate); however, they may possibly have diagnostic potential when incorporated in AAb assays to a panel of TAAs.

## 6. Autoantibodies to GIPC-1 Protein

The protein known as GIPC-1, a member of a family of PDZ-domain conserved proteins, is involved in regulation of G-protein signaling and is upregulated in breast and ovarian carcinomas [37, 83–85]. With use of 27.B1 and 27.F7 human monoclonal antibody specific to GIPC-1 protein, Yavelsky et al. [83] demonstrated a positive immunnohistochemical staining for GIPC-1, respectively, in 24/25 (96%) and 11/23 (48%) breast ductal carcinomas, 9/10 (90%) and 8/15 (53%) breast lobular carcinomas, 0/4 and 0/4 breast ductal carcinomas *in situ*, 0/4 and 0/4 breast lobular carcinomas *in situ*, 0/4 and 0/4 breast fibroadenomas, and 0/4 and 0/4 breast hyperplasias. GIPC-1 staining with 27.B1 and 27.F7 antibodies was positive only in invasive breast carcinomas (27.B1 displayed a higher reactivity rate than 27.F1) whereas GIPC-1 staining with 27.B1 and 27.F7 antibodies was negative in *in situ* and benign tumors (*P* < .001). The authors [83] assume that GIPC-1 protein is cancer-associated and hypothesize that serum AAbs to GIPC-1 may possibly serve as a marker for invasive breast carcinoma. With use of a novel technique of chemiluminescent optical fiber immunoassay (the instrument is called chemiluminescent optical fiber immunosensor), Salama et al. [37] tested sera from 22 breast carcinoma patients, 11 epithelial ovarian carcinoma patients, and healthy controls for the presence of IgM anti-GIPC-1 AAbs. The chemiluminescent optical fiber immunosensor detected 77% and 54% anti-GIPC-1 AAbs positive sera within breast and ovarian carcinoma patients, respectively, as compared to ELISA, which only detected 27% and 18%, respectively [37]. The authors conclude that the chemiluminescent optical fiber immunoassay is an efficient technique for prompt detection of AAbs to TAAs and, thus, foresee that the newly developed chemiluminescent optical fiber immunosensor might serve as an efficient tool for early diagnosis of breast and ovarian carcinomas [37].

## 7. Autoantibodies to c-myc and c-myb Protein

The myc gene encodes for a transcription factor, c-myc protein, that is essential for cell growth and proliferation and is broadly implicated in tumorigenesis [86–88]. Serum anti-c-myc AAbs were detected by ELISA in 12/94 (12.7%) patients with newly diagnosed breast carcinoma and 3/40 (7.5%) patients with ductal carcinoma in situ [23]. Positive seroreactivity was defined as an absorbance value greater than the mean plus two standard deviations of a normal cohort. The sensitivity and specificity were 13% and 97%, respectively, for breast carcinoma and 8% and 97%, respectively, for ductal carcinoma in situ. It has been concluded that measurement of serum AAbs to c-myc protein only is of little value for screening and early diagnosis of breast carcinoma; however, AAbs to c-myc may have promising diagnostic potential when incorporated in AAb assays against a panel of TAAs [23].

The Myb gene encodes for a transcription factor, c-myb protein, that is required during multiple stages of T cell development and is involved in cell cycle G1/S transition and antiapoptosis [89, 90]. Using Western blotting, Sorokine et al. [38] demonstrated that IgG AAbs against c-myb protein were present in the sera of 31/72 (43%) breast carcinoma patients compared to 12/49 (24.5%) healthy controls (*P* = .036). No significant correlation was observed between the presence of circulating AAbs to c-myb protein and the expression of the c-myb gene in breast tumors.

## 8. Autoantibodies to NY-ESO-1 Protein

Cancer-testis (CT) antigens are encoded by a group of genes predominantly expressed in human germline cells. They are downregulated in somatic adult tissues but may become aberrantly expressed in several types of cancers. CT antigens mapping to chromosome X are referred to as CT-X antigens and distinguished from non-X CT antigens located on autosomes [91, 92]. The CT-X antigens represent more than half of all CT antigens and their expression is frequently associated with a poorer outcome and higher grade and advanced stage tumors [91, 92]. Only few studies have explored the presence of CT antigens (NY-ESO-1 and/or MAGE-A) in breast carcinoma rendering contradictory results [93–95]. Interestingly, recent studies showed elevated expression of the CT antigens, NY-ESO-1 and MAGE-A, in triple-negative (ER-negative, PR-negative, and HER2-negative) breast carcinomas [91, 96].

Serum anti-NY-ESO-1 AAbs were identified by ELISA in 25/94 (26.6%) patients with newly diagnosed breast carcinoma and 3/40 (7.5%) patients with ductal carcinoma in situ [23]. Positive seroreactivity was defined as an absorbance value greater than the mean plus two standard deviations of a normal cohort. The sensitivity and specificity were 26% and 94%, respectively, for breast carcinoma, and 8% and 94%, respectively, for ductal carcinoma in situ. It has been concluded that measurement of serum AAbs to NY-ESO-1 protein only is of little value for screening and early diagnosis of breast carcinoma; however, AAbs to NY-ESO-1 may have promising diagnostic potential when incorporated in AAb assays against a panel of TAAs [23].

## 9. Autoantibodies to BRCA Protein

The wild-type BRCA genes, BRCA1 (located on chromosome 17q21) and BRCA2 (located on chromosome 13q12-13), are tumor-suppressor genes. The 185delAG and 5382insC mutations in the BRCA1 gene and the 6174delT mutation in the BRCA2 gene have been found to be significantly more common among Ashkenazi Jews (Jews of eastern European ancestry) (1 in 40, 2.5%) in comparison to the general population (1 in 800 to 1in 300, 0.12%–0.33%). Carriers of these “Ashkenazi mutations” have a significantly increased lifetime risk of breast carcinoma (about 50%), ovarian carcinoma and other carcinomas as compared to noncarriers [97].

AAbs to BRCA1 and BRCA2 protein were identified by ELISA in the sera of 8/94 (8.5%) and 32/94 (34%) invasive breast carcinoma patients, respectively, and 1/40 (2.5%) and 9/40 (22.5%) ductal carcinoma *in situ* patients, respectively [23]. Positive seroreactivity was defined as an absorbance value greater than the mean plus two standard deviations of a normal cohort. The sensitivity and specificity of evaluating serum anti-BRCA1 AAbs were 8% and 91%, respectively, for invasive breast carcinoma, and 3% and 91%, respectively, for ductal carcinoma *in situ*. The sensitivity and specificity of evaluating serum anti-BRCA2 AAbs were 34% and 92%, respectively, for invasive breast carcinoma, and 23% and 92%, respectively, for ductal carcinoma *in situ*. It has been concluded that (1) assessment of serum AAbs to BRCA1 protein alone is of no value for screening and early diagnosis of breast carcinoma, (2) serum AAbs to BRCA1 have no diagnostic potential even if incorporated in AAb assays against a panel of TAAs, (3) assessment of serum AAbs to BRCA2 protein alone is of little value for screening and early diagnosis of breast carcinoma, and (4) serum AAbs to BRCA2 may have, however, promising diagnostic potential when incorporated in AAb assays against a panel of TAAs [23].

## 10. Autoantibodies to Endostatin Protein

Endostatin, one of the most potent known natural inhibitors of angiogenesis, is a C-terminal fragment of collagen XVIII, which is highly expressed in the perivascular basement membrane of tumor-associated blood vessels [98, 99]. Elevated serum levels of endostatin have been found in metastatic cancer patients and have been correlated to the clinical course of the disease in various tumor types [39, 100].

Bachelot et al. [39] examined in 2006 with use of Western blotting approach the immunoreactivity of serum samples of breast carcinoma patients against recombinant human endostatin and found an inverse correlation between the incidence of naturally occurring serum antiendostatin AAbs and extent of disease. Serum antiendostatin AAbs were detected in 4/24 (16%) healthy women, 24/36 (66.6%) patients with localized breast carcinoma, and 25/59 (42.4%) patients with metastatic breast carcinoma. Differences were statistically significant between all breast carcinoma patients and healthy controls (*P* < .0001) and between localized and metastatic breast carcinoma patients (*P* = .03). The detection of serum antiendostatin AAbs was correlated to better survival in metastatic breast carcinoma patients. The median survival time of the 25 patients with detectable serum anti-edostatin AAbs was 20 months compared to 7 months for the other 34 patients (*P* = .03). There was no correlation to circulating levels of endostatin. The authors [39] have concluded that a natural immune reaction against endostatin can occur in breast cancer patients. The incidence of serum antiendostatin AAbs is higher in patients with localized disease and is associated with a better prognosis in patients with metastatic disease. It has been suggested that the reaction of anti-edostatin AAbs against tumor blood vessels may slow down tumor progression in breast carcinoma patients and partially explain the better survival of serum antiendostatin AAb-positive patients [39].

## 11. Autoantibodies to Lipophilin B Protein

Lipophilins and mammaglobins are members of the uteroglobin family, and mammaglobin has been shown to be highly breast tissue specific [101, 102]. More recently, lipophilin B, which is also present in breast tissue and other tissues, has been identified as forming a complex with mammaglobin that is potentially secreted into serum [103, 104].

Carter et al. [40] observed in 2003 that preexisting AAbs to lipophilin B peptide are absent in 20 healthy donor sera and 30 lung carcinoma sera, but present in the sera of 20/74 (27%) breast carcinoma patients overall, and 13/35 (37.1%) advanced-stage (stage IV) breast carcinoma patients. This immune response was different from that seen to recombinant mammaglobin and native mammaglobin-lipophilin B complex. The authors [40] have suggested that the humoral immune responses to lipophilin B may serve as a diagnostic indicator, particularly for breast carcinoma.

## 12. Autoantibodies to Cyclin Proteins

Cyclins are molecules that control the progression through the cell cycle. Cyclin B1 is important in the cell cycle progression from G2-to-M phase and has been shown to be overexpressed in several tumors [41, 105]. Several studies [3, 106] have shown that serum AAbs to cyclin B1 protein can be found in patients with various tumors and might be a useful diagnostic marker in combination with AAbs against several other tumor antigens. Suzuki et al. [41] examined by ELISA the presence of serum AAbs to cyclin B1 in 120 patients (7 with breast carcinoma, 17 with pancreatic cancer, 27 with colon cancer, and 69 with lung cancer) and found that the highest proportion of patients strongly positive (absorbance ≥ 1) for AABs to cyclin B1 was in the breast carcinoma group (3/7, 42.8%). A correlation of serum AAbs to cyclin B1 to higher level of tumor cyclin B1 expression was found. AAbs to cyclin B1 in patients with breast and colon carcinoma were primarily of the IgG isotype whereas patients with pancreatic and lung carcinoma had in addition AAbs of the IgA isotype. The authors [41] have speculated that anticyclin B1 AAbs may have a role as a serum marker for early detection of cancer.

Lu et al. [1] demonstrated by recombinant ELISA the presence of serum AAbs to cyclin D1 in 3/40 (7.5%) breast carcinoma patients compared to 4/80 (5%) healthy controls. It seems that AAbs to cyclin D1 protein have no diagnostic potential when examined alone; however, they may possibly have diagnostic potential when incorporated in autoantibody assays against a panel of TAAs.

## 13. Autoantibodies to Fibulin Protein

Fibulin-1(Fbln-1) is a member of an emerging family of glycoproteins found in extracellular matrix (ECM) and blood and has been observed to inhibit in vitro adhesion and motility of various carcinoma cell lines [107, 108]. Fbln-1 has been implicated as having a role in cancer, especially in breast carcinoma, and possible involvement in triggering protective antitumor immune responses [109–112].

With use of SEREX, serum AAbs to the glycoprotein Fbln-1 were identified in 15/20 (75%) breast carcinoma patients compared to 4/20 (25%) healthy controls (*P* < .0006) [42, 110]. It has been concluded that the SEREX-defined molecule Fbln-1 is able to elicit both cellular and humoral immune responses in breast carcinoma patients. The finding that Fbln-1 expression is associated with improved survival in patients with lymphoid infiltrate at the tumor site suggests the possible involvement of Fbln-1 in triggering protective antitumor immune responses [112]. In addition, it has been suggested that AAbs to Fbln-1 may perhaps be exploited as a tool for early detection of breast carcinoma [111].

## 14. Autoantibodies to Insulin-Like Growth Factor Binding Protein 2 (IGFBP-2)

The insulin-like growth factors, IGF-1 and IGF-2, the type 1 and type 2 IGF receptors, and the six known IGF binding proteins, IGFBP-1–6, comprise a complex system of peptide hormones, cell surface receptors and circulating factors involved in the regulation of growth, survival and differentiation in a vast number of tissues [113]. The IGFBPs are secretory proteins and possess an 80% sequence homology with each other, reserving binding preferences for either IGF-1 or IGF-2 [114]. IGFBPs have been proposed to have the following functions: (1) to act as transport carrier proteins of IGFs, (2) to stabilize and prolong the half-lives of IGFs thereby regulating their metabolic clearance, (3) to provide a means of tissue- and cell- type- specific localization, and (4) to directly stimulate or inhibit interactions of the IGFs with their receptors [114]. The IGFBP-2 is a 36 kDa secretory protein which, unlike other IGFBPs, is not glycosylated and occurs only in its nonphosphorylated form.

Lu et al. [1] demonstrated by recombinant ELISA the presence of AAbs to (IGFBP-2) in the sera of 21/142 (15%) breast carcinoma patients compared to 2/100 (2%) healthy controls. Goodell et al. [43] demonstrated by specifically designed his-tagged capture ELISA (based on lysate from genetically engineered Chinese hamster ovary cells) the presence of AAbs to IGFBP-2 in the sera of 4/80 (5%) breast carcinoma patients, 32/80 (40%) colorectal carcinoma patients and 2/200 (1%) healthy controls. These corresponded to a significant difference between all carcinoma patients and healthy controls (*P* = .008), between breast carcinoma patients and healthy controls (*P* = .032), and between colorectal carcinoma patients and healthy controls (*P* < .001) [43]. The authors concluded that the AAbs to IGFBP-2 assayed by capture ELISA can discriminate between cancer patients and controls [43]. It seems, however, that AAbs to IGFBP-2 have little value for screening and early diagnosis of breast carcinoma when examined alone. AAbs to IGFBP-2 may perhaps have diagnostic potential when incorporated in AAb assays against a panel of TAAs.

## 15. Autoantibodies to TOPO2*α* Protein

Topoisomerase II is a ubiquitous enzyme that regulates DNA under- and overwinding, and removes knots and tangles from the genome. Its two homologous isoforms, topoisomerase II*α* and topoisomerase II*β*, share ~70% amino acid sequence identity and display similar enzymatic activities. Topoisomerase II*α* (TOPO2*α*) is an essential enzyme, and its levels increase dramatically during periods of cell growth. It is tightly associated with mitotic chromosomes and has an essential role during DNA replication and mitosis [115].

Lu et al. [1] revealed by recombinant ELISA the presence of AAbs to TOPO2*α* protein in the sera of 8/115 (7%) breast carcinoma patients compared to 4/200 (2%) healthy controls. It seems that serum AAbs to TOPO2*α* have no diagnostic potential in breast carcinoma when examined alone; however, they may possibly have diagnostic potential when incorporated in autoantibody assays against a panel of TAAs.

## 16. Autoantibodies to Cathepsin D Protein

Primary biological function of enzymatically active cathepsin D is protein degradation in an acidic milieu of lysosomes [116]. Failure of this function resulted in accumulation of lipofuscin in variety of cell types, neurodegeneration, developmental regression, and visual loss. Procathepsin D, secreted from cancer cells, acts as a mitogen on both cancer and stromal cells and stimulates their proinvasive and prometastatic properties. Procathepsin D/cathepsin D levels represent an independent prognostic factor in a variety of cancers [116].

Using recombinant ELISA, Lu et al. [1] detected AAbs to cathepsin D protein in the sera of 5/100 (5%) breast carcinoma patients compared to 3/100 (3%) healthy controls. It seems that serum AAbs to cathepstin D have no diagnostic potential in breast carcinoma when examined alone; however, they may possibly have diagnostic potential when incorporated in autoantibody assays against a panel of TAAs.

## 17. Conclusions and Personal Viewpoints

Breast carcinoma is the most common malignancy and the most frequent cause of death from cancer in women. Traditional diagnostic tools for early detection, namely, manual breast examination, imaging studies (mammography, ultrasound, and MRI), and measurement of serum CA-15-3 are crippled with insufficient sensitivity and specificity. The mean sensitivity of mammography has been estimated to be 77% (range: 29%–97%) [117–119]. The rate of false-negative mammography has been reported to be 4%–34% [120, 121]. Serum AAbs to specific TAAs are detectable in cancer patients even when the tumor is obscured clinically. Evidently, the human immune system recognizes the autologous TAAs as “nonself” and makes a humoral immune response very early in the disease process. Thus, the identification of serum AAbs to TAAs could potentially be used as a novel tool for screening and early diagnosis of breast carcinoma. Nevertheless, it has been shown that measurement of AAbs against a single TAA is of little value and only assessment of AAbs to a tailor-made panel of TAAs may have promising diagnostic potential. The implications of this would be that AAbs to TAAs would provide a simple blood test for screening and early diagnosis of breast carcinoma. Nevertheless, it must be remembered that measurement of serum AAbs to TAAs for screening and early diagnosis of breast carcinoma is still investigational and should be carried out along with traditional screening and diagnostic studies. Our personal viewpoints regarding the management of women having a blood test for serum AAbs to breast carcinoma TAAs are as follows.

1. Women with mammography findings that are highly suggestive of breast carcinoma (BI-RADS category 5) or suspicious for breast carcinoma (BI-RADS category 4) [122] would require an immediate breast biopsy to obtain tissue for histological diagnosis irrespective of the status of serum AAbs to breast carcinoma TAAs.
2. In women with probably benign findings on mammography (BI-RADS category 3) [122], presence of serum AAbs to breast carcinoma TAAs would strengthen the decision to perform an immediate breast biopsy to obtain tissue for histological diagnosis rather than to wait six months for the next mammography. On the other hand, in women with probably benign findings on mammography (BI-RADS category 3), absence of serum AAbs to breast carcinoma TAAs would support the decision to wait six months for the next mammography.
3. In women with negative findings on mammography (BI-RADS category 1) and in women with benign finding(s) on mammography (BI-RADS category 2) [122], presence of serum AAbs to breast carcinoma TAAs might lead to a decision to perform immediate additional imaging studies (ultrasound and/or MRI) or, in women with BI-RADS category 2, even an immediate breast biopsy to obtain tissue for histological diagnosis rather than to wait one year for the next routine annual screening mammography. On the other hand, in women with negative findings on mammography (BI-RADS category 1) and in women with benign finding(s) on mammography (BI-RADS category 2) [122], absence of serum AAbs to breast carcinoma TAAs would support the decision to wait one year for the next routine annual screening mammography.
4. Women with an incomplete mammography assessment (BI-RADS category 0) [122] would need immediate additional imaging evaluation (ultrasound and/or MRI) and/or prior mammograms for comparison irrespective of the status of serum AAbs to breast carcinoma TAAs. Nevertheless, the presence of serum AAbs to breast carcinoma TAAs in such women would hasten the assessment by additional imaging studies and might even bring to the performance of an immediate breast biopsy to obtain tissue for histological diagnosis.

## Figures and Tables

**Table 1:** Frequency of identified circulating AAbs to TAAs in breast carcinoma.

AAb to TAA	Positive	Total	%	Comment	Reference
p53	22 6^a^	94 40^a^	23.4 15.0^a^	Promising diagnostic potential when incorporated in AAb assays to a panel of TAAs	[23]
p53	11	24	45.8	Association with higher risk for relapse.	[24]
p53	31	144	21.5	Correlation to higher stage, lymph node metastasis, negative ER, positive c-erbB-2 and worse survival.	[25]
p53	8	101	7.9	Correlated neither with p53 cytosolic assay nor with prognostic factors.	[26]
p53	22	220	10	Promising diagnostic potential when incorporated in AAb assays to a panel of TAAs	[1]
p53	296	2006	14.7	Summary of 15 studies (1979–1999). Frequency of AAbs: 2.8%–47.5%. Few studies showed association with high grade and poor survival.	[27]
MUC1	19 9^a^	94 40^a^	20.2 22.5^a^	Promising diagnostic potential when incorporated in AAb assays to a panel of TAAs.	[23]
MUC1	2	24	8.3	No correlation to circulating mucin levels or stage of disease.	[28]
MUC1	36^b^ 11^c^	140^b^ 61^c^	25.7^b^ 18.0^c^	Inverse correlation to extent of disease. Suggested role in protection against disease progression.	[29]
MUC1	20	100	20	Promising diagnostic potential when incorporated in AAb assays to a panel of TAAs.	[1]
HSP-27	219	579	37.8	Association with improved survival.	[30]
HSP-60	18 16^a^	58 49^a^	31 33^a^	Promising diagnostic potential.	[31]
HSP-90	46	125	36.8	Correlation to extent of disease. Promising diagnostic potential.	[32]
HSP-90	135	214	63.1	Association with higher mortality rate.	[33]
HER2/neu	16 5^a^	94 40^a^	17.0 12.5^a^	Promising diagnostic potential when incorporated in autoantibody assays against a panel of TAAs.	[23]
HER2/neu	11	20	55	HER2/neu oncoprotein elicits an immune response and may be used as a target for specific immunotherapy.	[34]
HER2/neu^b^	12 9^d^ 3^e^	107 44^d^ 63^e^	11.2 20.4^d^ 4.8^e^	Correlation to positive HER2/neu status in the primary tumor (*P* = .03).	[35]
HER2/neu^c^	3	45	6.6	Incidence is lower in advanced-stage disease compared to early-stage disease. Suggested role in limiting disease progression.	[36]
HER2/neu	30	225	13	Promising diagnostic potential when incorporated in AAb assays to a panel of TAAs.	[1]
GIPC-1	17	22	77	Promising diagnostic potential.	[37]
c-myc	12 3^a^	94 40^a^	12.7 7.5^a^	Promising diagnostic potential when incorporated in autoantibody assays against a panel of TAAs	[23]
c-myb	31	72	43	No correlation to c-myb status in the primary tumor.	[38]
NY-ESO-1/ LAGE-1	25 3^a^	94 40^a^	26.6 7.5^a^	Promising diagnostic potential when incorporated in autoantibody assays against a panel of TAAs	[23]
BRCA1	8 1^a^	94 40^a^	8.5 2.5^a^	No diagnostic potential, even if incorporated in autoantibody assays against a panel of TAAs	[23]
BRCA2	32 9^a^	94 40^a^	34.0 22.5^a^	Promising diagnostic potential when incorporated in autoantibody assays against a panel of TAAs	[23]
Endostatin	24^b^ 25^c^	36^b^ 59^c^	66.6^b^ 42.4^c^	Inverse correlation to extent of disease. No correlation to circulating levels of endostatin. Association with better prognosis in advanced-stage disease.	[39]
Lipophilin B	20 13^c^	74 35^c^	27.0 37.1^c^	Correlation to extent of disease. Promising diagnostic potential.	[40]
Cyclin B1	3	7	42.8	Correlation to higher level of tumor cyclin B1 expression. Questionable diagnostic potential.	[41]
Cyclin D1	3	40	7.5	Questionable diagnostic potential	[1]
Fibulin	15	20	75	Promising diagnostic potential.	[42]
IGFBP2	21	142	15	Questionable diagnostic potential	[1]
IGFBP2	4	80	5	Questionable diagnostic potential	[43]
TOPO2*α*	8	115	7	Questionable diagnostic potential	[1]
Cathepsin D	5	100	5	Questionable diagnostic potential	[1]

^a^Ductal carcinoma *in situ* (DCIS).

^b^Early-stage disease.

^c^Advanced-stage disease.

^d^HER2/neu-positive tumor.

^e^HER-2/neu-negative tumor.
